# A Multiscale Instance Segmentation Method Based on Cleaning Rubber Ball Images

**DOI:** 10.3390/s23094261

**Published:** 2023-04-25

**Authors:** Erjie Su, Yongzhi Tian, Erjun Liang, Jiayu Wang, Yibo Zhang

**Affiliations:** 1 School of Physics and Microelectronics, Zhengzhou University, Zhengzhou 450001, China; 2 Institute of Optoelectronic Information Science, Zhengzhou University, Zhengzhou 450001, China

**Keywords:** rubber ball cleaning system, image segmentation, feature fusion, polarized self-attention, Pyramid Vision Transformer, attention mechanism

## Abstract

The identification of wear rubber balls in the rubber ball cleaning system in heat exchange equipment directly affects the descaling efficiency. For the problem that the rubber ball image contains impurities and bubbles and the segmentation is low in real time, a multi-scale feature fusion real-time instance segmentation model based on the attention mechanism is proposed for the object segmentation of the rubber ball images. First, we introduce the Pyramid Vision Transformer instead of the convolution module in the backbone network and use the spatial-reduction attention layer of the transformer to improve the feature extraction ability across scales and spatial reduction to reduce computational cost; Second, we improve the feature fusion module to fuse image features across scales, combined with an attention mechanism to enhance the output feature representation; Third, the prediction head separates the mask branches separately. Combined with dynamic convolution, it improves the accuracy of the mask coefficients and increases the number of upsampling layers. It also connects the penultimate layer with the second layer feature map to achieve detection of smaller images with larger feature maps to improve the accuracy. Through the validation of the produced rubber ball dataset, the Dice score, Jaccard coefficient, and mAP of the actual segmented region of this network with the rubber ball dataset are improved by 4.5%, 4.7%, and 7.73%, respectively, and our model achieves 33.6 fps segmentation speed and 79.3% segmentation accuracy. Meanwhile, the average precision of Box and Mask can also meet the requirements under different IOU thresholds. We compared the DeepMask, Mask R-CNN, BlendMask, SOLOv1 and SOLOv2 instance segmentation networks with this model in terms of training accuracy and segmentation speed and obtained good results. The proposed modules can work together to better handle object details and achieve better segmentation performance.

## 1. Introduction

Condensing equipment is an indispensable large-scale heat exchange equipment in the electric power, petrochemical, and other industries. During long-term operation of the condenser, impurities in the cooling water will collect on the inner surface of the cooling tube, causing the inner wall of the cooling tube to scale and even block the cooling tube in serious cases. The rubber ball cleaning system is designed to clean and descale the condensing unit [1,2]. The rubber ball cleaning system mainly works by rubbing the inner wall of the rubber ball, which is larger than the diameter of the condensate tube. However, under the influence of friction conditions and water quality of cooling water, the rubber ball wears, the diameter decreases, and the phenomenon of ball leakage and ball loss occurs in the system, which reduces the cleaning effect. In order to achieve the best cleaning effect, we need to monitor the number and diameter changes of rubber balls in real time to replace the rubber balls in time and ensure the cleaning effect. This paper proposes an innovative image segmentation method based on neural networks, which can realize online identification of rubber balls, diameter detection, and reduce labour cost.

For rubber balls in cooling water, this paper adopts an image segmentation method to detect the diameter and number of rubber balls. In recent years, there has been much research on image segmentation in water, such as image segmentation based on fuzzy theory, which uses fuzzy nonlinear transformation to improve the image. J.C. Bezdek [3] proposed a fuzzy C-mean clustering image segmentation algorithm, which maximizes the similarity of intra-class data objects and minimizes the similarity of inter-class data objects. The algorithm is also able to retain the target information well. Yiru Wang et al. [4] proposed an improved Markov Random Field model for underwater image segmentation in 2015, which combines MRF model with Hard C-mean clustering. The combination of MRF model and Hard C-mean clustering technique can obtain the underwater image segmentation method and combines labelling and local features. Liu, Y. et al. [5] designed a fine segmentation model for underwater images in 2020 and proposed an improved level set image segmentation algorithm, which can accurately segment underwater images but is sensitive to light. Wei Wei et al. [6] proposed a local threshold segmentation method based on image grayscale waveform, applying asymmetric median filtering to remove noise and detect peaks and valleys on the gray wave to locate edges and entities, and this method can significantly improve the multi-noise and illumination of uneven images.

Due to the problems that rubber balls are mainly in cooling water, the target density of collected rubber ball images is large, the rubber balls will cover each other, fast movement, and uneven illumination, it is not easy for traditional machine learning image segmentation to capture the characteristics of rubber ball images; the image edges are easily expanded and distorted, and it will be a very tedious task to obtain the ideal segmentation effect. Therefore, this paper adopts deep learning based image segmentation. In 2017, Kai-Ming He [7] added mask prediction branch to the structure of Faster R-CNN [8] and improved ROI Pooling, proposed ROI Align, and implemented a new instance segmentation network. Mask R-CNN belongs to two-stage method: the first stage uses RPN (region proposal network) to generate ROI (region of interest) candidate regions; and the phase two model predicts the category, bounding box offset, and binarization mask for each ROI. After that, Zhaogin Huang et al. [9] proposed a network block to regress the instance features together with the corresponding predicted masks to the mask IOU. The mask scoring strategy calibrates the inconsistency between mask quality and mask scoring and improves the instance segmentation performance by prioritizing more accurate mask predictions during COCO AP evaluation. Li Bing et al. [10] improved the network structure based on the Mask R-CNN algorithm improvement network by first replacing the standard convolution of some residual modules in the ResNet feature extraction network with deformable convolution; then, they connected the spatial attention mechanism module and the channel attention mechanism module in parallel and added deformable convolution to these two modules, which significantly improved the instance segmentation accuracy, as well as the edge refinement. Zonghui Guo et al. [11] used the remote context capability of the transformer to capture the global information of underwater images and improve the underwater image information. Xu, Xi et al. [12] combined the multiscale transformer with CNN module and were able to extract the contextual information of different regions and the segmentation effect was effectively improved. Huang, Andi et al. [13] proposed a multiscale feature fusion network, based on the SSD network, and combined the backbone network with CBAM module so that the network focuses on the target information and has obvious advantages in underwater images with higher accuracy. However, the rubber ball is an image moving in water, and it is necessary to improve the segmentation speed while improving the segmentation accuracy. The YOLO network is a single-stage real-time target recognition model with fast recognition speed. According to the characteristics of this model, Bolya [14] proposed Yolact, a fully convolutional real-time instance segmentation model which implements a one-stage instance segmentation network; the results had excellent segmentation speed. Combining the above studies and the problems of rubber balls, this paper designs a multi-scale feature fusion instance segmentation model based on the attention mechanism. This model can segment the moving rubber balls under cooling water impact in real time while taking into account the segmentation accuracy. This method is based on the Yolact model: (1) it introduces Pyramid Vision Transformer (PVT) [15] in the backbone network to replace the convolution module, uses a spatial-reduction attention layer of the transformer to enhance the ability of feature extraction at different scales as well as spatial reduction to reduce computational cost, and fuses multi-scale features by feature pyramids to reduce the loss of semantic information and achieve the extraction of more detailed information; (2) Integrating Polarized Self-Attention (PSA) [16] and the feature fusion module to fuse image features across scales at the FPN [17] maintains high internal resolution by separating spatial and channel attention independently of each other, adds a non-linear component to fully retain high-resolution information, and improves the quality of feature maps at different scales; (3) The prediction module separates the mask branches separately and combines dynamic convolution with multi-convolution kernel fusion to enhance the model representation and improve the accuracy of the mask coefficients. The detection module performs intensive prediction on the extracted fusion features and uses the SIOU loss function [18] and Fast NMS to filter out the position of the object prediction frame, which improves the IOU computation rate and the target frame suppression accuracy.

## 2. Related Work

The rubber ball cleaning system is an environmentally friendly and effective cleaning method for industrial condensers, but there are problems such as the system losing balls, leaking balls, and the inability to replace the balls in time. In response to these problems, Japan’s Hitachi has proposed the BRM system using fibre optic sensors to automatically detect the number of recirculating cleaned balls. Germany’s Taprogge has proposed the BEM system using water flow to measure the effectiveness of rubber ball cleaning. Zhou Dengjin et al. [19] proposed a PLC based rubber ball online cleaning system which calculates the fouling thermal resistance online and can flexibly control the cleaning process to achieve energy saving. Han Jian [20] proposed a new type of rubber ball cleaning system for condenser cleaning and local accumulation of rubber balls, using numerical simulation to select the ball throwing method and PLC for obstacle detection. Li Zhi et al. [21] proposed an automatic detection device for rubber ball cleaning based on image processing, using image stitching, image pre-processing, and the minimum error segmentation method for image segmentation. This method is capable of detecting the number and radius of rubber balls, but has low detection efficiency for noisy images. In this paper, we propose an automatic monitoring system based on neural networks, mainly using deep learning image segmentation models for segmentation.

The real-time segmentation of cleaning rubber balls is a relatively novel problem, mainly because the cleaning rubber balls have high density, fast movement, and a light reflection issue. In the downsampling process of the traditional convolutional neural network, the feature representation ability is gradually degraded and the shallow semantic information is weak. As the convolutional layer is gradually deepened, the semantic information is enhanced, but the location information is weakened and the feature resolution is reduced, so it is necessary to improve the depth segmentation model on innovation. Wang Hua et al. [22] proposed an improved U-net model for underwater mineral segmentation by introducing the sampling module and the residual module on the pyramid into the U-net model to achieve superior segmentation performance. Yu Shengliang et al. [23] proposed a U-net segmentation model based on the mix transformer for underwater collision object recognition segmentation. They also introduced the OHEM cross-entropy loss function to improve the learning ability, which has good robustness to underwater images. To improve the accuracy of the model, attention mechanisms are embedded in the network to enhance the primary features and filter out the secondary features. Attention models were first introduced in the Natural Language Processing (NLP) domain through transformer models. Later, attention mechanisms were gradually used in computer vision. This was followed by self-attention research by Bello et al. [24]. These research initiatives have greatly benefited computer vision tasks such as image classification and semantic segmentation. Subsequently, Kumar et al. [25] proposed deformable attention blocks to enable more effective contextual information linking. Wang [26], Lu [27], and Kun Lu [28] et al. separately proposed an attention-based mechanism for segmentation networks, which also achieved good results. Therefore, we also introduce an attention mechanism based on the Yolact network. Here is an introduction to the Yolact network.

Given the real-time capabilities of the Yolact network, we adopt Yolact as the underlying model to solve the problem. The main idea of Yolact is to directly add the mask branch to the one-stage object detection algorithm without adding any ROI pooling operation. It divides the instance segmentation into two parallel branches: one for classification and one for mask prediction:

(1) Using FCN [29] to generate larger-resolution prototype masks which are not specific to any instance.
(2) The object detection branch adds an extra head to predict the mask factor vector for instance-specific weighted encoding of the prototype mask.

Finally, we take the instance of the target detection branch after Fast NMS, multiply the prototype mask and the mask factor vector one by one, and then, combine the multiplied results for output.

**Figure 1:**
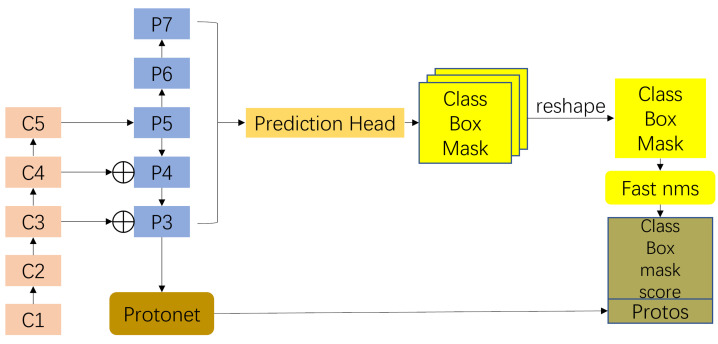
Architecture of Yolact, where C1∼C5 is the backbone network, P3∼P7 is the neck FPN module, whose output is connected to the prediction head, and the output of the prediction head is connected to the Detection Module. The output of the highest resolution layer P3 is connected to the instance generation module Protonet, which generates the instance mask.

The model structure of the Yolact network is shown in Figure 1. The model is mainly composed of the backbone network, prediction head, Fast NMS, Protonet [30], and the final image clipping and thresholding. The common feature backbone networks of Yolact model include VGG [31], ResNet [32], DenseNet [33], etc. DSSD [34] is used as the prediction head. First, the image features are extracted from the backbone; then, the Protonet branch generates the instance mask, the prediction head branch generates the mask coefficients, and the output results of the two branches are linearly multiplied to obtain the global mask; finally, the mask is clipped and thresholded to obtain the target mask.

## 3. A Multi-Scale Feature Fusion Real-Time Instance Segmentation Model Based on Attention Mechanism

**Figure 2:**
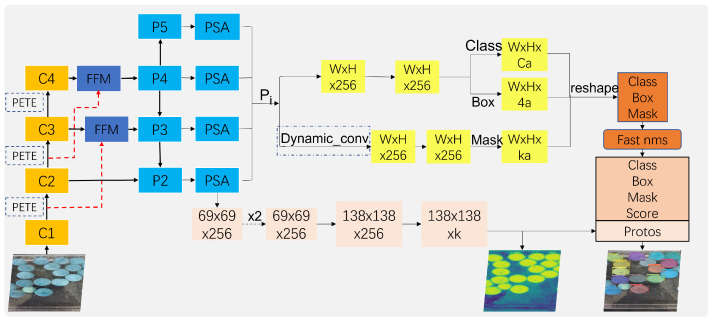
A multi-scale Yolact model based on attentional mechanisms, where C1∼C4 is the Pyramid Vision Transformer backbone module. Feature fusion module (FFM) integrates low-level and high-level feature information. Module P2∼P5 is the FPN module with polarized self-attention (PSA) module inserted between the FPN module and the prediction head, prediction head output of category confidence, position offset, mask coefficient, mask coefficient using dynamic convolution branching separately, and detection module using loss function SIoU and Fast NMS.

The specific model used in this paper is shown in Figure 2, which is an end-to-end single-stage instance segmentation model for real-time monitoring. Its basic modules and corresponding enhancements are described below.

### 3.1. Yolact Network

Yolact [14] is an efficient single-stage instance segmentation model that draws on the YOLO [35,36,37,38] series of object detection and is real-time. Compared with other instance segmentation models, Yolact focuses on speed and has been greatly improved. Therefore, the network framework of this paper is based on the Yolact network, where C1∼C4 is the Pyramid Vision Transformer (PVT) backbone network, and P2∼P6 is the neck FPN module with Polarized Self-Attention (PSA) module inserted between the neck FPN module and the prediction head, and prediction head output category confidence, position offset, mask coefficient, mask coefficient using dynamic convolution branching separately, and detection module using loss function SIOU and Fast NMS. The output of the highest resolution layer P2 output is connected to the instance generation module Protonet, which generates the instance mask. Comparing the above figure, we can see that our improvement is mainly in the feature backbone network module. The original convolutional layer is changed to the PVT module with dynamic weights and global receptive fields, as shown in the orange boxes in the figure above. To output high resolution feature maps and enhance the difference between target and background, the PSA is added to the output of the neck, as shown in the blue boxes in the figure. The Fast NMS speed of the prediction box is used, as shown in the brown box in the figure.

### 3.2. Pyramid Vision Transformer

**Figure 3:**
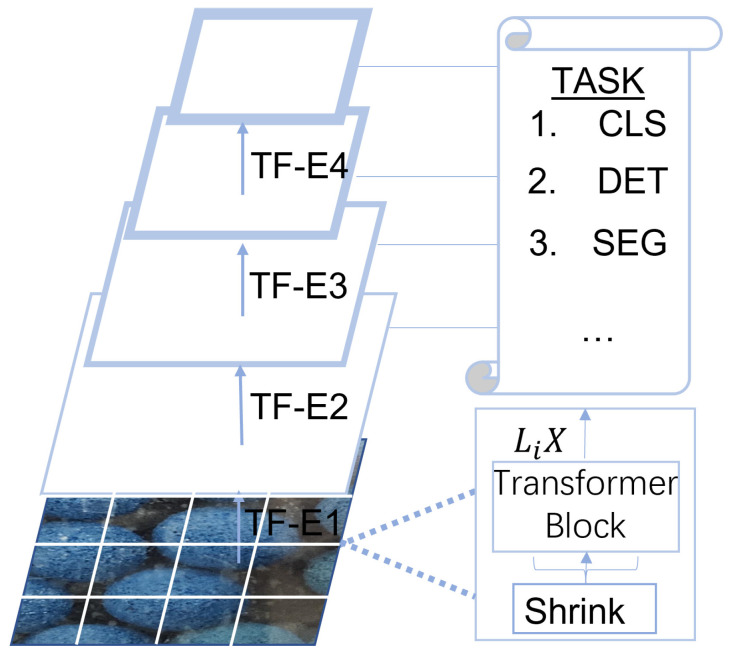
Pyramid Vision Transformer. Pyramid structure with four levels, each with a Patch Embedding and Transformer Encoder (PETE) module. Each layer replaces the convolution module with a transformer to obtain the global features of the image.

**Figure 4:**
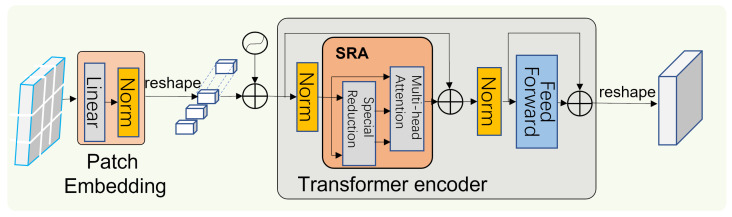
Patch Embedding and Transformer Encoder (PETE) Patch Embedding is mainly used for feature dimensionality reduction and Transformer Encoder for image feature encoding.

The PETE is in each layer of PVT. The structure of PVT is shown in Figure 3. Patch Embedding is mainly used for feature dimensionality reduction and Transformer Encoder is used for image feature encoding. The structure of Patch Embedding and Transformer Encoder is shown in Figure 4. We replaced the residual convolution module with a PETE module to reduce the loss of image features, followed by the design of a progressive pyramid structure and Spatially Reduced Attention (SAR), which can reduce resource consumption and allow the backbone network the flexibility to learn multi-scale and high-resolution features. The backbone network has four layers generating feature maps at different scales. Compared to the previous layer, the dimension of each layer is halved, and the resolution of the feature map is reduced to obtain a multi-scale feature map. Each layer has a similar structure, Patch Embedding Layer and Transformer Encoder. Patch Embedding is mainly used to encode image information, and the Transformer Encoder is used to decode and enrich feature information. For the first layer, given an input image of W×H×3, first tokenize it, that is, perform Patch Embedding, and divide it into $\frac{W\times H}{4^{2}}$ patches, that is, the size of each patch is 4×4×3. The dimension of the feature map finally obtained by this layer is halved, and the number of tokens is correspondingly reduced by four times. After that, the feature map of each layer is $\frac{1}{4},\frac{1}{8},\frac{1}{16},\frac{1}{32}$. Since the patch dimensions of each layer are different, they correspond to different position vectors. The position vector is calculated as follows:

(1) $$\begin{array}{c} {PE}_{\left(pos,\;2i\right)}=\sin\left(pos/{10,000}^{2i/d}\right)\\ {PE}_{\left(pos,\;2i+1\right)}=\cos\left(pos/{10,000}^{2i/d}\right) \end{array}$$

where pos represents the position of the patch in the image, and *d* represents the dimension of the patch. Next Transformer Encoder includes SRA and Feed Forward. SRA is a variant of MHA composed of multiple self-attentions. The modification of the multi-head attention mechanism is mainly to reduce the amount of computation. Since the number of image tokens in each layer is different, the higher the number, the more computation, and the calculation amount of self-attention depends on the length of the input sequence. The Attention function can be described as mapping a query and a set of key–value pairs to an output, where the query *Q*, the key *K*, the value *V*, and the output are all vectors. The output is computed as a weighted sum of values, where the weight assigned to each value is computed by the query compatibility function with the corresponding key. The output matrix is computed as follows:

(2) $$\mathrm{Attention}\left(Q,K,V\right)=\mathrm{softmax}\left(\frac{QK^{T}}{\sqrt{d_{k}}}\right)V$$

where $d_{k}$ is the number of columns for Q,K. Multi-Head Attention allows the model to jointly focus on information from different representation subspaces at different locations,

(3) $$\mathrm{Multihead}\;\left(Q,K,V\right)=\mathrm{Concat}\;({\mathrm{head}\;}_{1},\;{\mathrm{head}\;}_{2},\ldots ,\;{\mathrm{head}\;}_{h})$$

(4) $$\begin{array}{c} {\mathrm{head}}_{i}=\mathrm{Attention}\;\left(QW_{i}^{Q},KW_{i}^{W},VW_{i}^{V}\right) \end{array}$$

where Concat is the concatenation operation, $W_{i}^{Q}\in R^{C_{i}*d_{\mathrm{head}}}$, $W_{i}^{W}\in R^{C_{i}*d_{\mathrm{head}}}$, $W_{i}^{V}\in R^{C_{i}*d_{\mathrm{head}}}$, and $W^{Q}\in R^{C_{i}*C_{i}}$ is a linear projection parameter, and its calculation amount is positively correlated with the spatial scale of Q,K, and *V*. Spatial-Reduction Attention performs spatial reduction operations on K and V before performing the attention operation to reduce the dimensions of *K* and *V*. SRA details are as follows:

(5) $$SR\left(X\right)=\mathrm{Norm}\left(\mathrm{Reshape}\left(X,R_{i}\right)W^{S}\right)$$

where *X* is the input sequence and $R_{i}$ is the spatial reduction rate. $W^{S}\in R^{R_{i}^{2}c_{i}\times C_{i}}$ is a linear projection, reducing the input dimension to $C_{i}$. $\mathrm{Reshape}(X,R_{i})$ reshapes the input sequence *X* dimension into $\frac{H_{i}W_{i}}{R_{i}^{2}}\times \left(R_{i}^{2}C_{i}\right)$. Therefore, the complexity of the computational inner product of SRA entering Attention is reduced from $H_{i}W_{i}$ to $\frac{H_{i}W_{i}}{R_{i}^{2}}$, and its computational cost is lower than that of MHA. The larger the value of $R_{i}$, the lower the computational cost is very obvious.

### 3.3. Improved FPN in the Model

**Figure 5:**
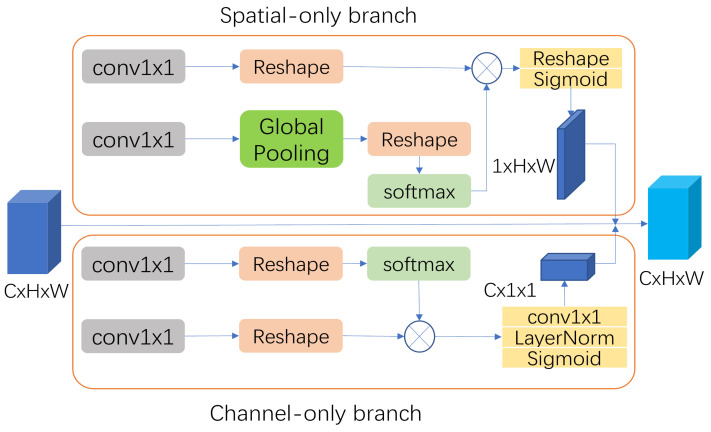
The polarized self-attention block under the parallel layout. The input features are passed through the spatial attention module and the channel attention module, which are placed in parallel, and finally multiplied linearly with the input feature map to output the result.

Maintaining high image resolution and preserving global information in fine-grained tasks such as image segmentation is critical to improving accuracy. Long distance information transmission in deep networks with multiple downsampling leads to weakened semantic information and reduced image resolution. This requires enhancement of the input–output long-range dependency modelling of high-resolution images, and we therefore introduce a polarized self-attentive (PSA) as well as a feature fusion module (FFM) [39] in the FPN module. The PSA emphasizes spatial and channel features, maintaining high internal resolution in channel and spatial attention calculations while fully collapsing the input tensor along the corresponding dimension; it also incorporates non-linearity in the attention mechanism consistent with fine-grained regression outputs. The PSA module uses the channel attention mechanism along the channel axis to highlight, as far as possible, the classification in which the pixel is located. The spatial attention mechanism is used from a spatial perspective to detect the location of the same semantic pixel and concatenate them to generate a valid feature descriptor. The PSA module is instantiated in Figure 5.

Through these operations, the PSA module helps to clean the rubber ball images by filtering the information to obtain salient features, which are then subjected to softmax normalization to expand the dynamic range of attention. A sigmoid function is then used for dynamic mapping and is capable of reducing the loss of detail.

**Figure 6:**
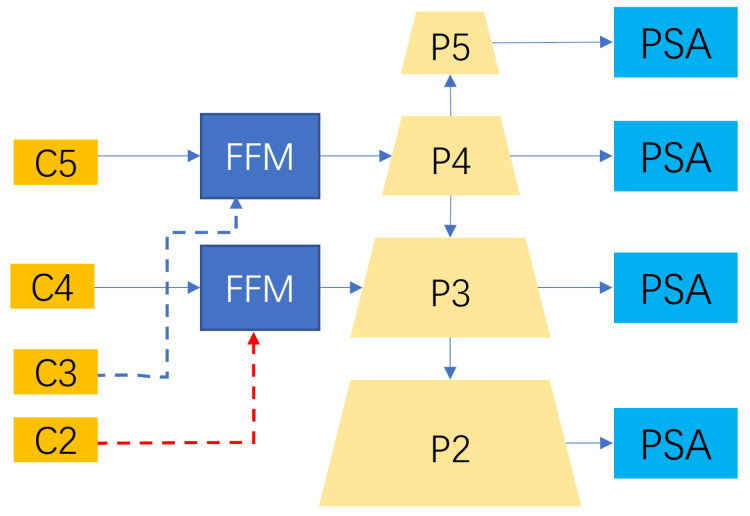
Improved FPN. Improved FPN based on feature fusion module and attention mechanism module PSA.

In the FPN model, feature maps are extracted at different scales. The lower level feature maps have less semantic information but rich spatial features; the higher level features are rich in semantic information but weak in spatial information. We use the FFM fusion of deep and shallow feature information to enhance the semantic information at the lower levels as well as the resolution at the higher levels. Thus, the segmentation performance is better improved. We combine the PSA module at the output of the FPN module to compensate for the loss of features at different scales. The highest resolution feature maps have the richest feature information, and the PSA module reduces redundant complex background information and false positives. This improvement can highlight the large amount of spatial and channel information in multi-level feature maps, which can improve prediction accuracy. The structure diagram of improved FPN module is shown in Figure 6.

### 3.4. Prediction Head in Model

**Figure 7:**
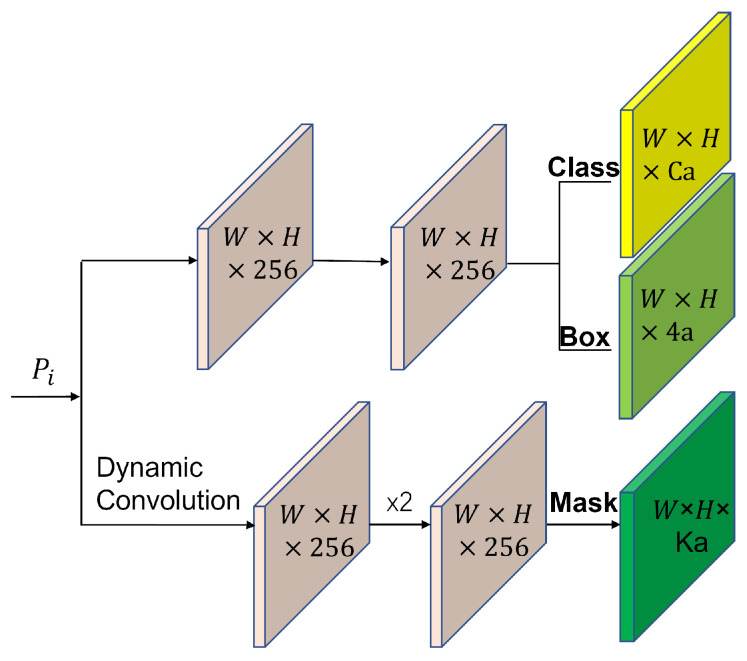
Prediction head architecture and dynamic convolution. In contrast to the Yolact prediction head, we separate the mask coefficient branch separately and use dynamic convolution to improve their accuracies.

The Yolact prediction head generates three types of output, namely category confidence, position offset, and mask coefficients. In the experiments, the mask accuracy was much lower than the target recognition accuracy, resulting in poor segmentation results. Therefore, we separate the mask coefficient branch on its basis and add dynamic convolution to improve the accuracy of the mask coefficients, slightly sacrificing the computational effort to improve the mask accuracy. Its structure is shown in Figure 7.

Dynamic convolution is the fusion of multiple convolution kernels in a deep network to improve model representation without increasing the depth and width of the network. Dynamic convolution pays attention to the convolution kernel, generates different weight coefficients for multiple convolution kernels, multiplies the weight coefficients with the corresponding convolution, and sums them to obtain the final convolution weight, which is then matrix multiplied with the input to obtain the output. The mathematical expression for dynamic convolution is as follow:

(6) $$\begin{array}{c} y=g\left({\tilde{W}}^{T}\left(x\right)x+\tilde{b}\left(x\right)\right)\\ \tilde{W}\left(x\right)=\sum_{k=1}^{K}\pi_{k}\left(x\right){\tilde{W}}_{k},\tilde{b}\left(x\right)=\sum_{k=1}^{K}\pi_{k}\left(x\right){\tilde{b}}_{k}\\ 0\leq \pi_{k}\left(x\right)\leq 1,\sum_{k=1}^{K}\pi_{k}\left(x\right)=1 \end{array}$$

where dynamic convolution outputs: *y*, $\pi_{k}$: indicate attention weights; aggregate weights: $\tilde{W}\left(x\right)$; and aggregation bias: $\tilde{b}\left(x\right)$. In addition, we increase the number of upsampling layers and connect the penultimate layer to the second layer feature map to achieve detection of smaller images with larger feature maps and improve the accuracy.

### 3.5. SIoU Loss Function in Model

The loss functions in the instance segmentation task are generally classification loss, detection loss, and segmentation loss. In the Yolact network, the loss function adds mask loss and prototype loss. IoU is the intersection ratio of candidate bounding boxes and ground truth bounding boxes, representing the overlap of the generated candidate bounding boxes with the original ground-truth bounding boxes. The conversion of the loss function of the bounding box from smooth L1 loss to IoU loss is a process of directly using the metric as the loss function. The mathemaatical expression of IoU in the model is as follows:

(7) $$IoU=\frac{\left|B\cap B_{gt}\right|}{\left|B\cup B_{gt}\right|}$$

where *B* is the prediction box and $B_{gt}$ is the true box. Corresponding loss function: $L_{IoU}$ = 1−IoU. However, if the predicted frame and the real frame do not intersect, the loss function is always one, the loss function is not controllable, and the gradient cannot be returned. Second, the sizes of the two predicted frames are the same, the two IoUs are also the same, and the IoU loss function cannot distinguish between them. The intersection situation is different. GIoU [40] introduces a penalty cost by adding a minimum circumscribed rectangle to wrap any two rectangular boxes; DIoU [41] considers the overlap area and center point distance, and CIoU [41] increases the aspect ratio factor of the prediction box. However, the orientation mismatch between the detection box and the ground truth box may cause the prediction box to fail to match the ground truth box during the training process, resulting in a worse model. The SIoU loss function [18] we use takes into account the angle vector between the bounding box regressions, making the predicted box move to the nearest axis fairly quickly. Subsequent methods only require a regression of coordinates X or Y, effectively reducing the total number of degrees of freedom. In order to obtain angle perception, the LF component is introduced, which is defined as follows:

(8) $$\begin{array}{c} \Lambda =1-2*\sin^{2}\left(\arcsin\left(x\right)-\frac{\pi }{4}\right)\\ x=\frac{c_{h}}{\sigma }=\sin\left(\alpha \right)\\ \sigma =\sqrt{{\left(b_{c_{x}}^{gt}-b_{c_{x}}\right)}^{2}+{\left(b_{c_{y}}^{gt}-b_{c_{y}}\right)}^{2}}\\ c_{h}=\max\left(b_{c_{y}}^{gt},b_{c_{y}}\right)-\min\left(b_{c_{y}}^{gt},b_{c_{y}}\right) \end{array}$$

where σ is the distance from the center point, α is the lower angle between the real frame $b^{gt}$ and the predicted frame *b*; $b_{c_{x}},b_{c_{y}}$ are the predicted frame center coordinates; $b_{c_{x}}^{gt},b_{c_{y}}^{gt}$ are the real frame center coordinates.

In this training model, we use a target box regression loss function SIoU that takes into account the distance between box centers, overlapping regions, aspect ratios, and angles, effectively reducing degrees of freedom, faster convergence, and more accurate inference in the training phase.

## 4. Experiments and Results

### 4.1. Dataset and Experimental Environment

**Figure 8:**
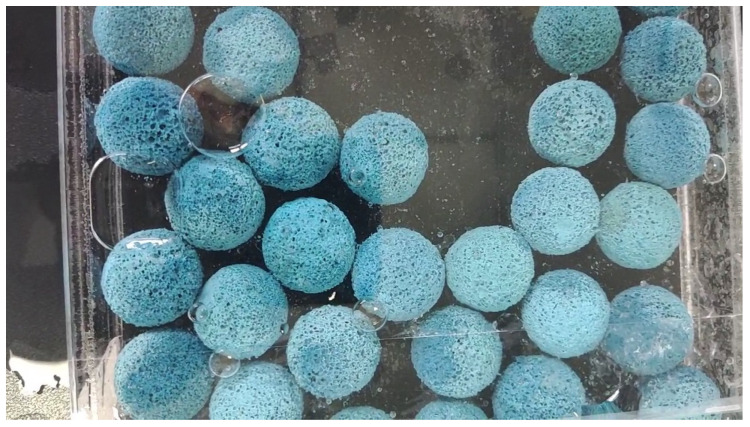
Sample image of cleaning rubber ball, with blisters, impurities etc.

The dataset used in our experience was the images randomly collected during the running process of the rubber ball to reflect the running state of the rubber ball and the volume change. The dataset was collected during the operation of the rubber ball cleaning system of the large condenser. The main equipment used are the special tube at the inlet end of the ball collector in the rubber ball cleaning device, which is easy to collect. The ball data were designed by CAD drawing. High-speed line scan cameras and lighting equipment were also used. The data collected by the camera had a frame rate of approximately 154 frames per minute. The frame rate collected by the camera must not be less than the flow rate of the water. Otherwise, the rubber balls in the collected images will be deformed. The dataset established in this paper has a total of 1000 images of size 2048 × 2000, including images with water environment and without water environment. After adaptive scaling, the images are unified into 550×550. Some sample images are shown in Figure 8.

LabelMe software was used to label the images. Since the rubber balls had the same appearance and were spherical, they were labelled with circles. On the collected data images, the labelling target is a rubber ball with a shape larger than a semicircle, which is convenient for the counting of the rubber balls. The dataset is in the COCO dataset format. The experiments were conducted using an NVIDA 1080Ti accelerated graphics card and the network development framework was Pytorch. The experiments were evaluated using the COCO dataset evaluation metric, which focuses on IoU, average accuracy mAP, AP, and fps to evaluate the network performance. In our experiments, we used the k-means clustering method to preset the size of the anchor box for the dataset to improve the detection performance.

### 4.2. Generate Anchor Box

**Figure 9:**
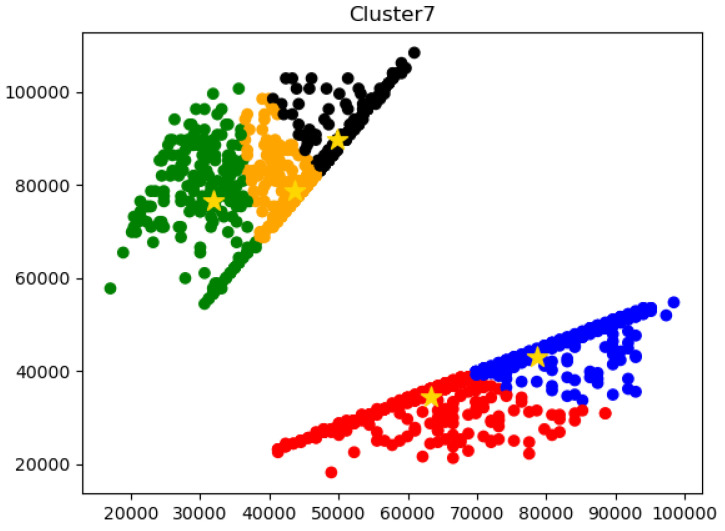
Clustering results. The horizontal and vertical axes represent the width and height of the rectangular box, respectively. The center of the cluster of five colors represents the anchor that fits the dataset better.

In the object detection segmentation algorithm, the manually designed anchor may not fit the dataset well; thus, in this article, we used the k-means algorithm clustering based on the genetic algorithm instead of the manual method to cluster the bounding box of the training set. We used the maximum IoU mean value of anchor and box as the index to automatically and vividly fit the anchor of the dataset and improve the network detection accuracy. We set five clusters and calculated the distance of each sample to the cluster centre. We updated the cluster centres several times to obtain the best clustering result, i.e., the most appropriate anchor. The clustering results are shown in Figure 9.

### 4.3. Ablation Experiment

In order to illustrate the advantages of the proposed algorithm, we compare its segmentation accuracy and speed with other modules. The dataset used in this paper is a continuous frame, which meets the requirements of the tracking algorithm. We set the batch size to 8 and the learning rate to 0.001, using equal intervals to adjust the learning rate and a decay multiplier of 0.1. The PVT module used in this paper improves the feature representation while reducing the loss of semantic information. To verify the capacity of the module, ablation experiments were performed to demonstrate this. The experimental results for different backbone networks in the rubber dataset are shown in Table 1. We compared the Mask R-CNN, BlendMask, SOLOv1, and SOLOv2 instance segmentation networks with this model in terms of training accuracy and segmentation speed. The comparison metrics are mAP and segmentation frame rate FPS for different target sizes are shown in Table 2.

**Figure 10:**
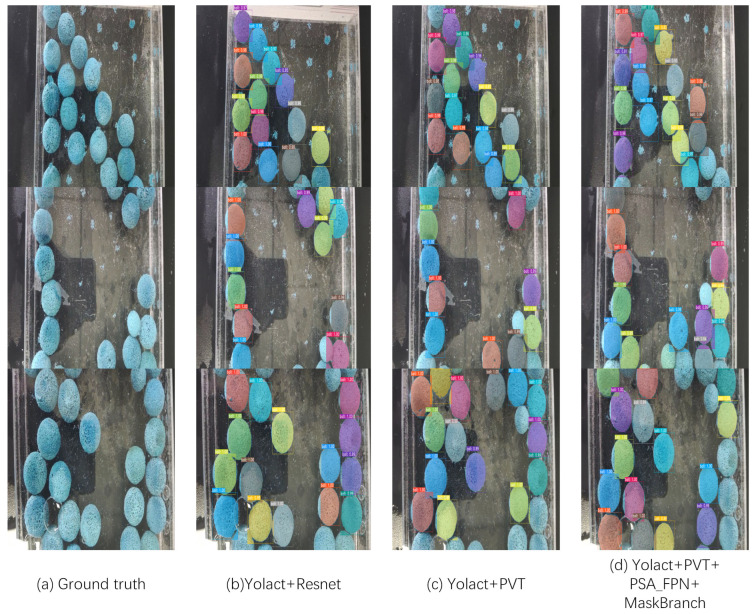
Visualization of segmentation results. (**a**), (**b**), (**c**) and (**d**), respectively, represent the segmentation results of different network improvements. The first column shows three different sample images, and the second, third, and fourth columns show the segmentation results under different modules. Each row represents the segmentation results of the model based on different modules for the same sample.

**Figure 11:**
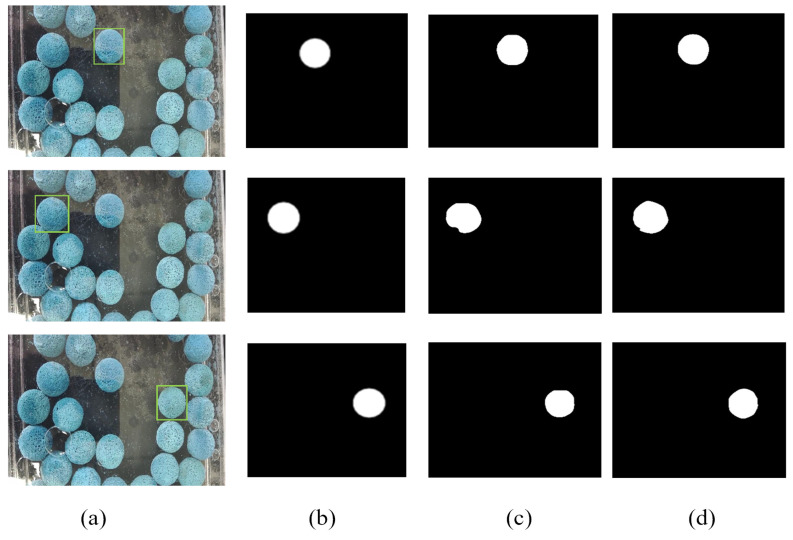
Visualization of mask results. (**a**) is the original rubber ball images, (**b**) is ground truth (GT), (**c**) is the result of the yolact network segmentation mask, (**d**) is the results of our model segmentation mask.

As can be seen from the table above, our network has approximately 10% better accuracy than the base network, while the segmentation speed is slightly reduced. This leads to the conclusion that the module we used can further improve the segmentation performance. Figure 10 and Figure 11 also give a more visual comparison of the images. From the visualization of the segmentation results and the segmentation mask results, our model has a higher segmentation accuracy, the mask edges are more detailed, and the segmentation exceeds that of the base model.

### 4.4. Analysis of Network Model Test Results

**Figure 12:**
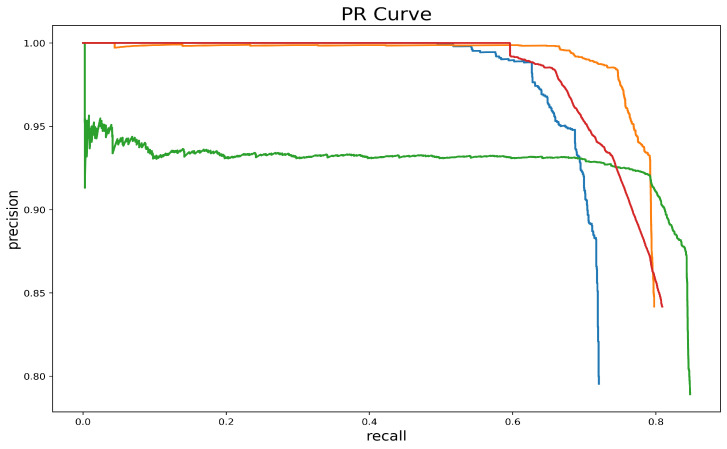
Comparison of PR curves. The green curve corresponds to the PR curve of the yolact network, the blue curve corresponds to the PR curve of Yolact + PVT; the red curve corresponds to the PR curve of Yolact + PVT + PSA_FPN; and the orange represents the PR curve corresponding to the model in this paper.

In order to qualitatively analyze the network segmentation performance, based on the characteristics of the studied data and the correlation between the evaluation metrics, we chose the PR curve, Dice coefficient, and Jaccard similarity coefficient for quantitative evaluation. A PR curve is a curve that represents the relationship between Precision and Recall. $Precision=\frac{TP}{TP+FP},Recall=\frac{TP}{TP+FN}$. The performance of the network model can be judged by the area under the PR curve: the larger the area, the better the network performance. As can be seen in Figure 12, our network performs better than the base network.

Another metric, the Dice coefficient, is used to evalute the effectiveness of the image segmentation method. It is the degree of overlap between the foreground pixels of the segmented image and the region of the ground truth foreground pixels, which is calculated as follows:

(9) $$Dice=\frac{2\times \mathrm{Recall}\times \mathrm{Precision}}{\mathrm{Precision}+\mathrm{Recall}}$$

The higher the value of the Dice coefficient, the better the performance of the model.

The Jaccard similarity coefficient, used in this paper to compare the similarity and difference between a finite set of samples, indicates the similarity between the foreground pixels of the segmented image and the ground truth foreground pixel region. The higher the Jaccard coefficient, the better the performance of the model segmentation. The Jaccard coefficient is calculated as follows:

(10) $$\begin{array}{ccc} \mathrm{Jaccard}\;(R,G) & = & \frac{R\cap G}{R+G-R\cap G}\\ & = & \frac{TP}{TP+FP+FN} \end{array}$$

*R* indicates the actual predicted outcome and *G* indicates the true truth. True Positives (TP), False Positives (FP), and False Negatives (FN) are used in this paper to calculate Jaccard coefficients.

**Figure 13:**
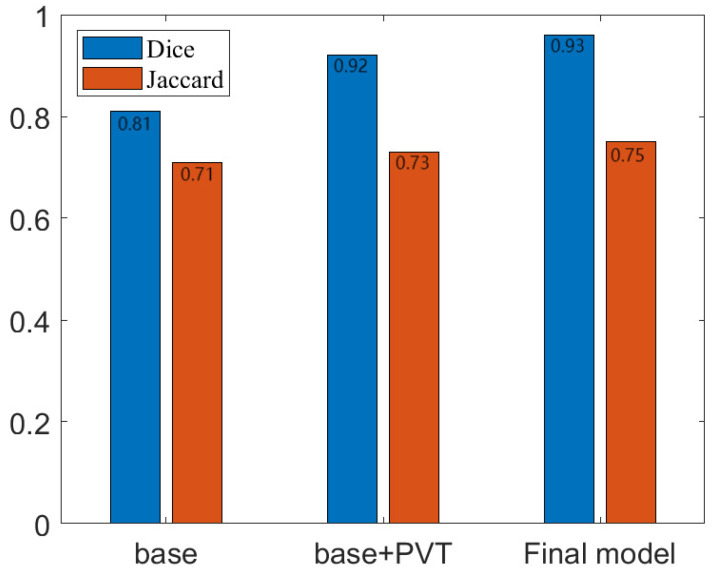
Jaccard coefficients and Dice scores for different models. The Jacobi coefficients and Dice scores for the three models were calculated and compared in a histogram format, with the specific values indicated.

As can be seen from Figure 13, the PETE-based PVT module we used improved the Dice score of the base network by about 3.3% and the Jaccard coefficient by about 3.4%. Our overall improved network improved the Dice score of the base network by about 4.4% and the Jaccard coefficient by about 4.5%. In addition, we also verified the average prediction accuracy under different IoU thresholds, and the results are shown in Table 3. Experimental results show that our network has a significant performance improvement in rubber dataset segmentation. The PETE module used in the paper, an improved FPN module based on the attention mechanism, and enhanced mask branches improve the segmentation performance of the base network. A backbone network based on the PETE module extracts image features at different scales to reduce feature loss; an improved FPN module better fuses multi-scale features and uses an attention mechanism to improve output resolution; an enhanced mask prediction branch provides further improvements in mask accuracy; an improved prediction head improves prediction accuracy. We propose these improved modules to enable the overall model segmentation performance to be improved.

## 5. Discussion

In the article, the backbone network uses the transformer module, which is demanding on the dataset and suffers from stability; thus, we use pre-trained weights. Our model increases the number of parameters compared to the base model, and although the segmentation accuracy is improved, the speed is slightly reduced.

## 6. Conclusions

A multi-scale feature fusion instance segmentation model based on the attention mechanism is proposed to address the problems of industrial condenser descaling rubber ball images with impurity, bubbles, and low real-time segmentation. The PETE module of the backbone network has multi-scale global feature extraction capability, and the FFM module fuses deep and shallow features to better extract image features; the improved FPN module integrates the PSA attention mechanism, which combines spatial and channel attention to further improve the network performance without increasing the number of parameters; the prediction head separates the mask branches separately. Combined with dynamic convolution, it improves the accuracy of the mask coefficients, increases the number of upsampling layers, and connects the penultimate layer with the second layer feature map to achieve detection of smaller images with larger feature maps to improve the accuracy; in the feature fusion module, we increase the number of sampling layers on the pyramid to improve the resolution of the feature map, increase the ability to extract small target features, and improve the accuracy of small target segmentation. According to the experimental comparison, our model can achieve 79.3% segmentation accuracy and 33.6 segmentation speed in the descaling rubber ball image segmentation task. The average precision of Box and Mask can also meet the requirements under different IOU thresholds. Combining real-time segmentation performance and segmentation accuracy, the proposed model outperforms Yolact, DeepMask, Mask R-CNN, BlendMask, SOLOv1 and SOLOv2. From the above, it can be concluded above that our proposed model has better segmentation effect in the real-time detection of rubber balls for condenser descaling, and there is room for further research in this work. For example, the model can also be applied to other segmentation tasks with the potential to further improve the accuracy.

## Figures and Tables

**Table 1:** Comparison of the performance of different module segmentation. A network model based on different modules is compared with the base model to highlight the advantages of this model.

Model	Box (%)		
	${\mathrm{AP}}_{\mathrm{all}}$	mAP	FPS
Yolact + VGG	61.97	28.2	33.1
Yolact + Darknet	62.53	28.7	34.4
Yolact + Resnet	68.02	29.3	35.7
Yolact + PVT	71.99	32.5	35.3
Yolact + PVT + PSA_FPN	73.14	33.2	34.3
Final model	79.30	36.7	33.6

**Table 2:** Performance comparison of different model segmentation. The improved model of this paper is compared with some classical instance segmentation models under the same experimental conditions.

Model	Box	Mask
Mask R-CNN	35.7	4.8
BlendMask	41.3	22.3
SOLOv1	30.8	32.5
SOLOv2	33.1	35.7
Yolact	28.2	37.1
final model	36.7	35.2

**Table 3:** The average prediction of different IOU thresholds. The average precision rates of the prediction frame and segmentation mask were calculated for ten IoU thresholds in the experiment.

	AP	Box	Mask
IoU			
all		78.11	72.74
0.50		92.10	92.10
0.60		90.11	90.11
0.65		90.11	90.11
0.70		90.11	90.11
0.75		89.10	89.10
0.85		86.07	81.71
0.90		68.61	51.44
0.95		29.24	9.65

## Data Availability

Not applicable.

## Sample Availability

All data for the current study can be obtained from the authors, but consent is required.
